# Expression of S100B during embryonic development of the mouse cerebellum

**DOI:** 10.1186/1471-213X-7-17

**Published:** 2007-03-15

**Authors:** Sabira Hachem, Anne-Sophie Laurenson, Jean-Philippe Hugnot, Catherine Legraverend

**Affiliations:** 1 Institut de Génomique Fonctionnelle, Département d'Oncologie Cellulaire et Moléculaire, 141, rue de la Cardonille, F-34094 Montpellier Cedex 5, France; 2 Unité INSERM 583, Institut des Neurosciences de Montpellier, 80, rue Augustin Fliche, 34091 Montpellier Cedex 5, France

## Abstract

**Background:**

In the cerebellum of newborn *S100B*-EGFP mice, we had previously noted the presence of a large population of S100B-expressing cells, which we assumed to be immature Bergmann glial cells. In the present study, we have drawn on this observation to establish the precise spatio-temporal pattern of *S100B *gene expression in the embryonic cerebellum.

**Results:**

From E12.5 until E17.5, S100B was expressed in the primary radial glial scaffold involved in Purkinje progenitor exit from the ventricular zone and in the Sox9+ glial progenitors derived from it. During the same period coinciding with the primary phase of granule neuron precursor genesis, transient EGFP expression tagged the Pax6+ forerunners of granule precursors born in the cerebellar rhombic lip.

**Conclusion:**

This study provides the first characterization of S100B-expressing cell types of the embryonic mouse cerebellum in a high-resolution map. The transient activation of the S100B gene distinguishes granule neuron precursors from all other types of precursors so far identified in the rhombic lip, whereas its activation in radial glial precursors is a feature of Bergmann cell gliogenesis.

## Background

The medial and lateral compartments of the alar plate of the metencephalon have been shown to confer distinct patterning information during neurogenesis and gliogenesis of the embryonic cerebellum (Cb) (for a review see [1]). Whereas cerebellar inhibitory neurons like Purkinje cells (PCs) and Bergmann glial cells are generated in the medial portion of the fourth ventricle (V4) [2], primary precursors of granule neurons (GPs) are produced from its lateral recesses, the so-called cerebellar rhombic lip (RL) [3]. During the third week of gestation in the mouse, radial glial cells of the medial aspect of the ventricular zone (VZ) progressively retract their somata towards the cortex and actively divide to generate precursors of Bergmann glial cells and astrocytes [4]. At the same time, young PCs migrate radially from the neuroepithelium to the surface in a strictly caudal-to-rostral order, paralleling the emergence of cohorts of neuron precursors from the RL and their superficial migration along the dorsal surface of the Cb primordium [5].

Using a line of transgenic mice in which tamoxifen-inducible Cre expression at the time of birth results in permanent β-galactosidase labeling, RL precursors destined to populate the internal granule layer (IGL) were estimated to be generated in a rostral-to-caudal sequence between E13 and E17 [6]. At the time, these Lac-Z-labelled IGL cells were thought to represent a homogenous population of granule neurons. A recent study has provided evidence that besides granule cells, the cerebellar RL gives birth to another IGL population of neurons, unipolar brush cells (UBCs), between E14.5 and E19.5 [7]. Furthermore, granule and UBC neurons are preceded by glutamatergic Deep Cerebellar Nuclei (DCN) neurons. The first DCN neuron precursors are born in the RL at around E10. At E11.5, they stream over the entire dorsal surface of the Cb, and from E12.5 until E14.5 they aggregate in the NTZ, a transient zone of differentiation [6,8,9].

The RL therefore appears to be highly dynamic, giving rise to distinct neuronal populations, and a consensus has now emerged from all these fate-mapping studies, which redefines the RL as a functional rather than anatomical entity. The RL is now considered as a territory within rhombomere 1 which is required for the sequential generation of all cerebellar and extracerebellar superficial migratory streams, therefore contributing neurons to the proprioceptive/vestibular/auditory sensory network which task is to sense the organism's position in space [8]. The cells of this functional system all depend on the expression of the basic-helix-loop-helix transcription factor Math1for their genesis [3], and the paired and homeodomain containing transcription factor Pax6 for their proper migration [10].

In *S100B*-EGFP mice, we had previously noted that the transgene is activated during histogenesis of the Cb between E13.5 and P3, and reported the existence of a large population of S100B+ cells in the Cb cortex of newborn mice [11]. Because S100B is commonly used as a marker of Bergmann glia and white matter astrocytes in the Cb of adult mice [12], we assumed that its presence in the embryonic Cb marked their precursors. S100B is a small EF-hand calcium and zinc binding protein, highly expressed in the adult vertebrate central nervous system (CNS) along with five other S100 family members [13]. The S100B protein sequence is extremely well conserved (> 97%) among mammals, suggesting that it is endowed with important physiological functions [14]. However, as judged by the vitality of mice strains lacking the S100B protein, there must be a fundamental resiliency of the developmental program involving the S100B protein [15,16].

S100B is a highly soluble protein implicated in the initiation and maintenance of a pathological, glial-mediated pro-inflammatory state, and its presence in biological fluids is a well-established biomarker for severity of neurological injury and prognosis for recovery [17]. A consensus sequence for S100B target proteins was published as (K/R)(L/I)xWxxIL and matches a region in the actin capping protein CapZ [18]. Several additional S100B targets are known, including p53[19], two NDR kinases [20], the RAGE receptor [21], protein kinase C, and Gap-43 [22,23]. The range of effector proteins so far identified suggests roles in the regulation of transcription, cell-cycle progression [24], and cell morphology. In astrocytes where S100B is abundantly expressed, its best-characterized roles involve modulating protein-protein interactions of all three classes of cytoskeletal structures, and preventing these interactions blocks astrocyte stellation [25].

With the present study, we sought to determine the precise spatio-temporal pattern of S100B expression in the embryonic Cb using the *S100B*-EGFP mouse as a model. We found that S100B protein expression in radial glial cells of the medial portion of the cerebellar VZ marks the onset of gliogenesis. In addition, we provide evidence for transcriptional activation of the endogenous *S100B *gene being associated with the prenatal phase of GP production in the RL.

## Results

### S100B expression in the cerebellum (E17.5)

**Figure 1:**
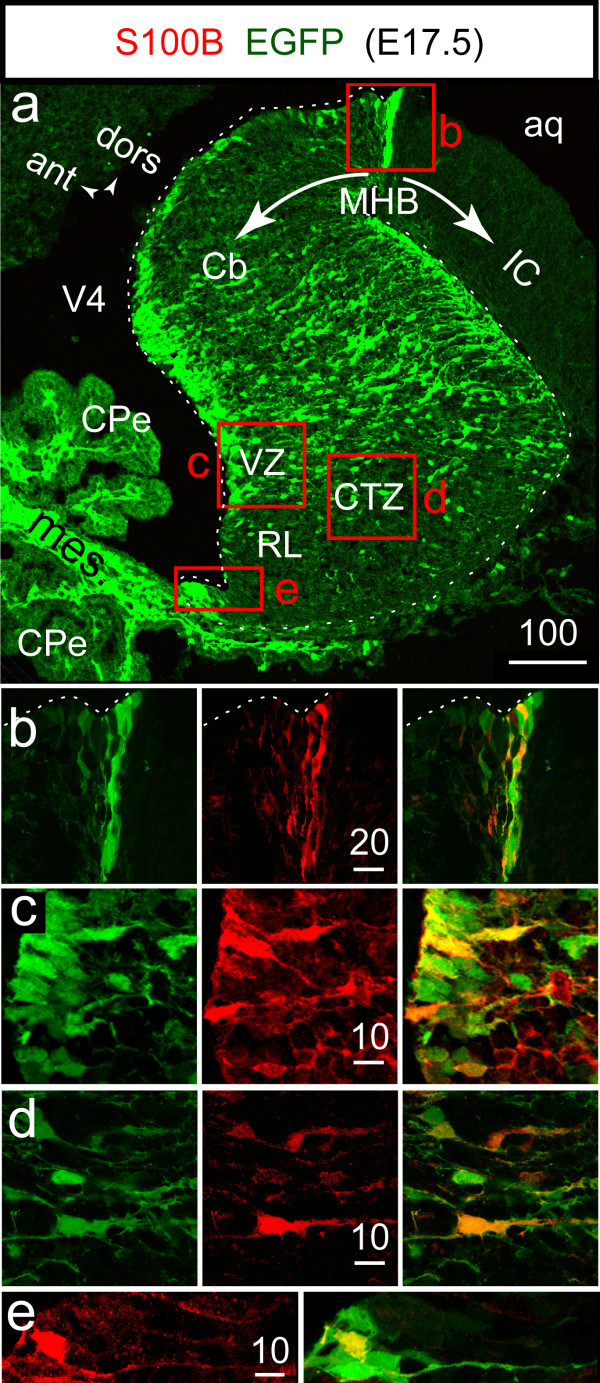
***S100B *gene driven expression of EGFP in S100B+ cells of the ventricular and cortical transitory zones of the cerebellum at E17.5**. **A**: confocal fluorescent image of a parasagittal section of the E17.5 *S100B*-EGFP cerebellar vermis. In addition to neural cells, the EGFP reporter is strongly expressed in the mesenchyme underlying the CPe. The staining patterns for S100B and EGFP are overlapping near the MHB (**B**), in the VZ (**C**), CTZ (**D**), and RL (**E**). The white dashed lines mark the ventricular and pial limits of the Cb. In this and the following figures, numbers above bars indicate the scale in microns.

The observation that incited us to launch the present study is illustrated in figure 1. At around E17.5, and compared to other regions of the brain, a relatively high level of the *S100B*-EGFP reporter protein was found in the Cb primordium (Fig 1A), and this was matched by a fair level of S100B protein expression in densely packed radial glial cells located at the midbrain-hindbrain boundary (Fig 1B, MHB) or bordering the fourth ventricle (Fig 1C), in isolated cells of the cortical transitory zone (Fig 1D), and to a lesser extent in the RL (Fig 1E). Unlike EGFP, both nuclear and cytoplasmic, the S100B signal was often restricted to the cytoplasm but specificity was inferred from the total absence of S100B immunosignal in corresponding sections obtained from S100B null embryos (data not shown).

Between E13.5 and E17.5, migratory Purkinje progenitors are subject to "contact guidance", a process by which they adhere to radial glial processes to reach their final destination in the cortex [26]. From E14.5 until P7, Bergmann glial cells are thought to derive from primary radial glial cells that translocate their somata from the VZ into the Purkinje cell layer, giving rise to a secondary radial glial scaffold [27]. Detection of S100B in the cerebellar radial glial scaffold at E17.5 therefore incited us to go back in time and determine exactly when, and in which cell type, transcription of the S100B gene is activated in the embryonic Cb.

### S100B expression in the cerebellar radial glial scaffold (E14.5–E16.5)

**Figure 2:**
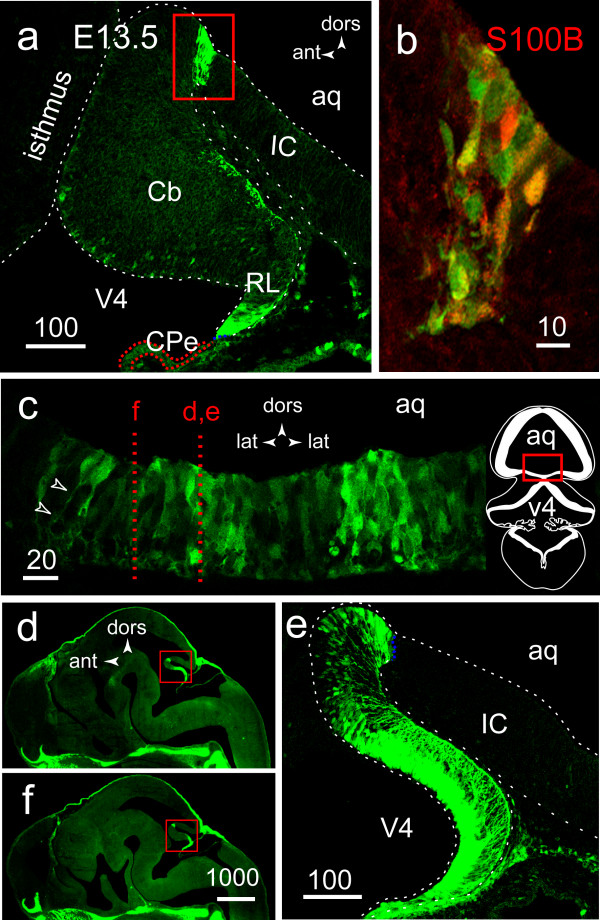
**pattern of *S100B *gene expression in the cerebellum and inferior colliculus before midline fusion of the cerebellar plates (E13.5)**. **A**: lower power confocal image of the Cb primordium, illustrating the strong EGFP signal present near the MHB (red box) in a parasagittal section. **B**: zooming on the boxed area in A reveals the high level of S100B/EGFP colocalisation at the single cell level. **C**: near the MHB, and on coronal sections, EGFP tags a stretch of neuroepithelial cells approx. 300 μM in width, emitting thin processes towards the pial surface (arrowheads). The red dotted lines represent the approximate planes of sections D and F. The glial scaffold (boxed area) is entirely **(D, E) **or only partially visible **(F)**, depending on how close to the midline is the plane of section. **E**: higher magnification of the boxed area in (D) illustrating the pattern of EGFP expression near the midline: both the S-shaped radial glial scaffold of the Cb, and the abutting IC neuroepithelium, are strongly labeled.

Compared to E17.5, the number of EGFP+ VZ cells co-expressing S100B was much reduced at E13.5, except in the narrow strip of cells located at the midbrain-hindbrain boundary (compare the boxed areas in Figs. 1A and 2A,B). To better understand the 3-dimentional structure of this region, we examined coronal sections as well and found that EGFP expression was prominent in a medial stretch of ventral neuroepithelium of approximate width 300 microns that belongs to the inferior tectal neuroepithelium, and not to the Cb as originally thought (Fig 2c). In the Cb, the number of EGFP+ cells was strikingly increased near the midline and organized in an S-shaped intensely EGFP+ radial glial scaffold spanning the primordium from the VZ to the pial surface (Fig. 2E). The scaffold could only be visualized in its integrity in paramedian sections (compare Figs 2D and 2F).

**Figure 3:**
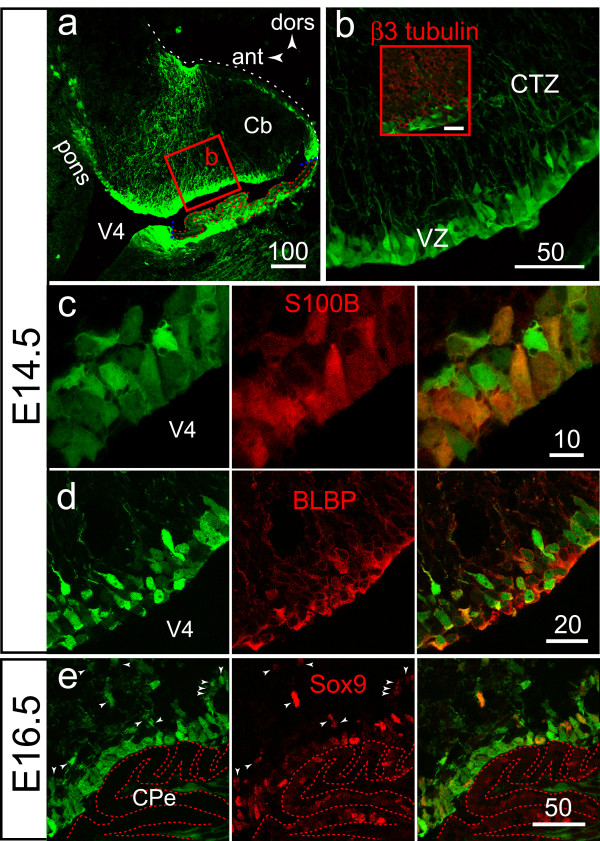
**S100B and EGFP are co-expressed in the radial glial scaffold of the cerebellar plates (E14.5 and E16.5)**. **A**: lower power view of the lateral portion (future hemisphere) of the E14.5 cerebellum plate. **B**: higher power view of the boxed area in **(A)**, illustrating localization of cell somata in the VZ and long radial processes traversing a CTZ filled with post-mitotic β3-tubulin+ neuron precursors (inset). **C**: zooming on the VZ area reveals a high level of S100B/EGFP colocalisation at the single cell level. **D**: As expected for radial glial cells, EGFP+ cells present in the VZ express BLBP. **E**: at E16.5, and in addition to radial glial cells and CPe cells, Sox9 expression is maintained in isolated cells emigrating from the VZ (arrowheads).

Figure 3A illustrates the general pattern of EGFP expression observable on parasagittal sections of a Cb hemisphere at E14.5. The radial glial nature of the cells aligned in the VZ was first inferred from the oval-shaped somata and long ascending processes traversing the entire thickness of the Cb primordium filled with post mitotic β 3 tubulin+ neurons (Fig 3B, inset). The radial glial nature of the cells was confirmed using the technique of immunohistochemistry to detect the expression of two markers: brain lipid binding protein (BLBP), a member of the large family of hydrophobic ligand binding proteins exclusively expressed in radial glia during embryonic brain development [28], and Sox9, a transcription factor expressed in the neuroepithelium and choroid plexus epithelium [29]. The high level of EGFP;S100B and EGFP;BLBP co-expression is illustrated in figures 3C, and 3D, respectively. As expected, we found that epithelial cells of the choroid plexus, radial glial cells and isolated cells located in the CTZ also expressed the transcription factor Sox9 from E13.5 (not shown) to E16.5 (Fig 3E).

Together these results indicated that before midline fusion of the Cb plates, EGFP and S100B were co expressed in the Sox9+ BLBP+ primary radial glial scaffold traversing the Cb primordium from the IC posterior limit to the RL, with a higher level of expression near the midline. In addition, EGFP;S100B co-expression was prominent in a median stretch of inferior tectal neuroepithelium directly abutting the rostral Cb primordium.

### Characterization of the *S100B*-EGFP cell population of the CTZ (E13.5–E16.5)

**Figure 4:**
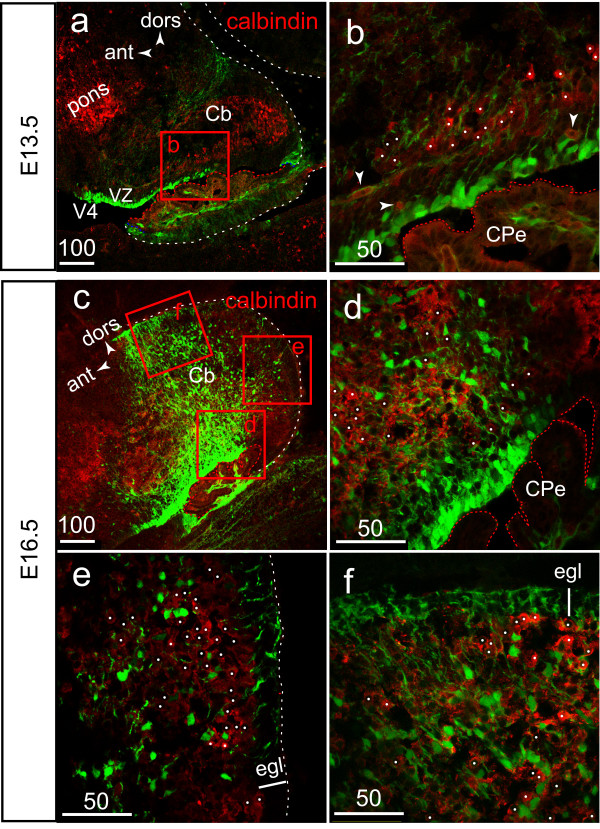
**EGFP is not expressed in post-mitotic calbindin+ Purkinje progenitors (E13.5–E16.5)**. **A**: lower power view of the lateral portion (future hemisphere) of the E13.5 Cb plate illustrating the broad subcortical distribution of calbindin+ PCs. **B**: higher power view of the boxed area in (A), illustrating the contacts between post-mitotic calbindin+ EGFP-negative PC precursors (white dots) and EGFP+ radial glial processes. Arrowheads point to the non specific red fluorescence of small capillaries. **C**: at E16.5, the radial glia-derived EGFP+ cell population is greatly increased. **D-F**: no matter which region of the Cb primordium is examined, the EGFP+ and calbindin+ populations are clearly separate entities. Most EGFP+ cells are connected to the pial surface via their apical process, constituting the so-called secondary radial glial scaffold used by the EGL population of GPs during their postnatal phase of radial migration.

In the mouse, all cerebellar PCs are produced during only three embryonic days from E10.5 to E12.5 [30], and most, if not all glial precursors in the Cb take the form of radial glial cells from E12.5 to E14.5, during the phase of PC migration [27]. Using an antibody directed against calbindin-1 (spot 35), which starts being expressed in post-mitotic PCs [26], we found no colocalisation of calbindin and EGFP from E13.5 until E16.5 (Fig. 4). Beginning on E13.5, EGFP expression was restricted to the single cell layer of radial glia bordering V4, whereas calbindin+ PCs were distributed in a broad cellular cortical zone of the future hemisphere (Fig. 4A). Some PCs located near the VZ were closely apposed to EGFP+ radial processes (Fig. 4B), which is a characteristic feature of migrating progenitors. The number of EGFP+ cells was greatly increased at E16.5 but EGFP and calbindin were never co-expressed in the same cells (Fig. 4C–F).

Based on the evenly distribution and morphology of EGFP+ cells connected to the pial surface via their apical radial process (Fig. 4E,F), which characterize prospective Bergmann glial cells [31], we conclude that the radial glia-derived EGFP+ population present in the Cb primordium between E13.4 and E17.5 likely contains glial precursors some of which are already contributing to the secondary Bergmann radial glial scaffold that will later be used by GPs to migrate from the EGL to the IGL.

### Characterization of the *S100B*-EGFP cell population of the RL (E12.5–E15.5)

**Figure 5:**
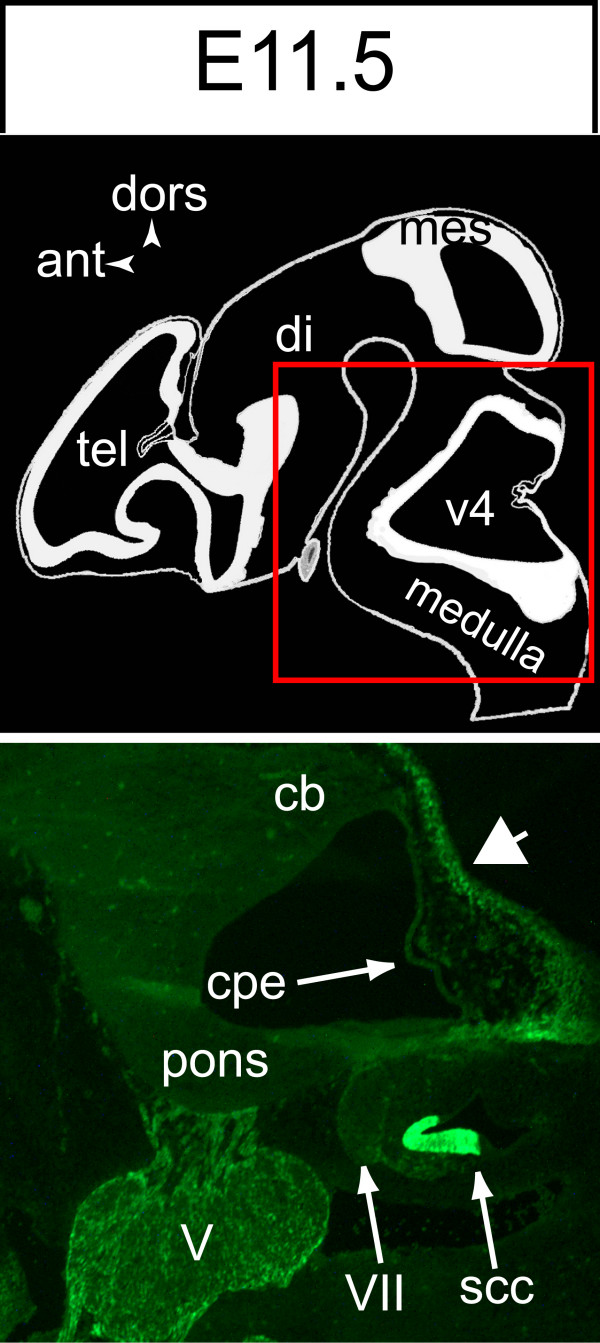
**EGFP is not expressed in the cerebellar rhombiclip before E12.5**. A lower power view of a future Cb hemisphere at E11.5 illustrates the absence of EGFP expression in the rhombic lip, contrasting with its presence in the neighbouring mesenchyme **(arrowhead)**, posterior semicircular canal **(SCC) **of the otic vesicle, and glial cells of the trigeminal **(V) **and facial **(VII) **ganglions.

**Figure 6:**
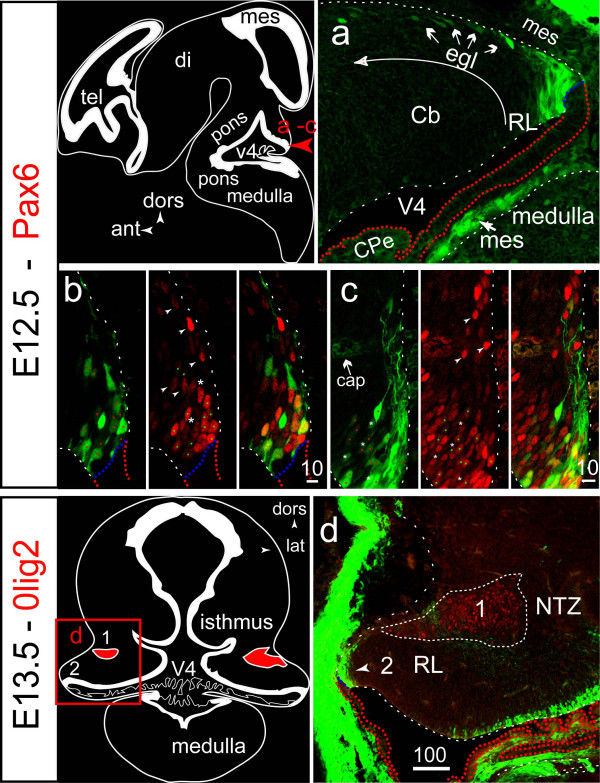
**The RL generates EGFP+ cells that are not precursors of DCN neurons (E12.5–E13.5)**. **A**: lower power view of a future Cb hemisphere at E12.5 illustrating the pattern of EGFP expression in two seemingly related cell populations: tightly packed bipolar cells in the RL, and isolated cells with a unipolar shape and a leading process contacting the pial surface in the nascent EGL, which is characteristic of migrating GPs (arrows). **B, C**: higher power views of the E12.5 RL, illustrating the localisation of EGFP+ Pax6+ cells in the medial **(B)**, and lateral **(C)**, portion of the RL, and the presence of Pax6+ EGFP- cells in the nascent EGL (arrowheads). Nuclei of the cells in which EGFP/Pax6 co-expression is obvious are marked by colored dots, EGFP+ Pax6- cells by asterisks. The long branching processes emitted by the RL EGFP+ cell population are better visualized in C. **D**: at E13.5 and on coronal sections, the Olig2+ NTZ (1) and EGFP+ EGL (2) constitute clearly distinct populations.

Before E12.5, EGFP expression was not detected in the RL (Fig 5), or any other territory of the CNS. Beginning on E12.5, EGFP expression was detected in a subpopulation of bipolar cells present in the RL (Fig 6A,B) and emitting subpial branching processes into the nascent EGL (Fig 6C). However, in contrast to cells present in the medial portion of the cerebellar VZ, BLBP expression was much reduced and expression of the transgene was not matched by detection of the S100B protein (data not shown). With some exceptions [32], it is now generally accepted that the RL generates exclusively neuronal precursors. Precursors of DCN glutamatergic projection neurons are born in the prospective RL and migrate into the future EGL before gathering in the NTZ between E10 and E13.5 [8,9], whereas the first cerebellar GPs and UBCs are born during the last gestational week beginning on E13 [7]. Therefore, we assumed that EGFP expression in the RL beginning on E12.5 could reflect activation of the S100B gene in GP or (and) UBC precursors, but not in DCN precursors.

The non DCN nature of EGFP+ RL precursors was tested by performing immunohistochemistry with a pair of antibodies directed against the transcription factor Pax6 expressed in the RL throughout its neurogenetic interval [10], and the transcription factor Olig2 known to label the NTZ starting on E12.5 [33]. The high proportion of EGFP+ Pax6+ double positives present in the lateral portion of the RL at E12.5 is illustrated in figures 6B and 6C (colored dots). Their non-DCN nature was inferred from the discontinuity that existed at E13.5, between the NTZ already filled with Olig2+ nuclei and the territory of EGFP expression restricted to the RL and posterior EGL (Fig 6D).

**Figure 7:**
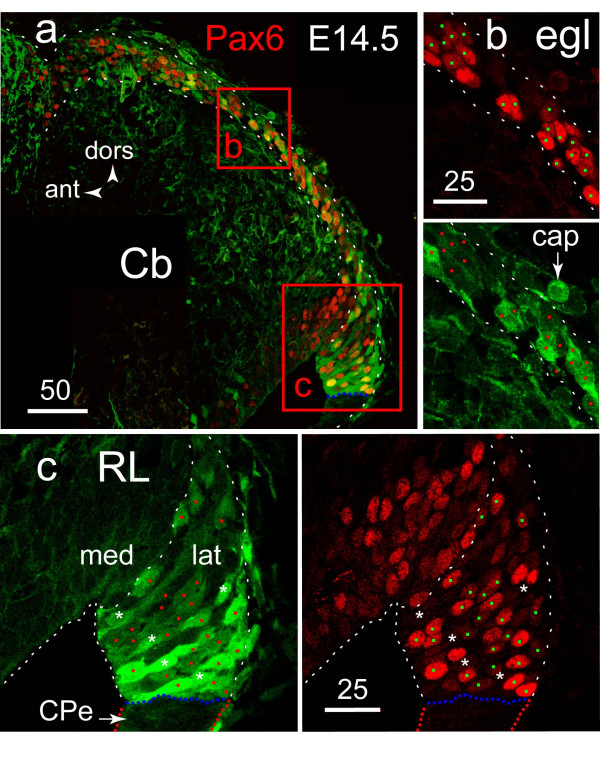
**EGFP+ Pax6+ granule precursors in the RL and EGLat E14.5**. **A**: lower power view of the lateral portion of the future Cb hemisphere, illustrating the pattern of EGFP/Pax6 co expression in the RL and EGL at E14.5. **B**: higher power view of the EGL showing clusters of migrating Pax6+ EGFP+ neuron precursors with their intensely Pax6+ nuclei (upper panel) and EGFP+ cytoplasm (lower panel). **C**: higher power view of the RL illustrating the predominant population of EGFP+ Pax6+ cells (colored dots) and the presence of EGFP+ Pax6- cells (asterisks).

Until E14.5 (Fig 7), a high level of EGFP expression was maintained in a subpopulation of Pax6+ precursors present in the RL and in the EGL, whereas from E15.5 (data not shown) onwards (Fig 1A, Fig 4C–F), EGFP expression was down-regulated in the EGL, and the number of EGFP+ cells present in the RL decreased considerably in proportion to the growth of the EGL. During that time, expression of the transgene was not matched by a significant S100B signal (data not shown). However, the possibility that the transgene could be ectopically expressed was discredited once we realized that S100B mRNA had previously been detected in that region [34] (high resolution pictures of the E14.5 stage available [35]).

From our results and the results provided by a recent study that will be discussed below [7], we believe that transient expression of the *S100B*-EGFP transgene is a specific marker of the primary phase of GP genesis in the embryonic RL.

## Discussions and conclusions

The aim of the present study was to establish the spatio-temporal pattern of S100B expression in the mouse embryonic Cb. We compared the distribution of the *S100B*-EGFP reporter to that of the endogenous S100B protein from E12.5 to E17.5. Our results establish the S100B protein as a marker of the BLBP+ Sox9+ primary radial glial scaffold starting on E13.5. In addition, and although the S100B protein was not detected, we provide evidence that *S100B*-EGFP expression in Pax6+ derivatives of the RL marked the onset of primary GP production. In addition, our results suggest that depending on the site of S100B synthesis, primary radial glial scaffold and derivatives or RL and neuron precursors, S100B may be retained within the cell or secreted.

### The onset of S100B expression in the cerebellar ventricular zone: marking the transition from neurogenesis to gliogenesis in primary radial glial cells

Although we found no mention of the S100B protein being expressed in the mouse embryonic cerebellar radial glia, the *S100B *mRNA was detected in the rat cerebellar radial glia [12]. Additionally, in a study designed to analyze the expression pattern of 158 murine orthologs of genes identified on human chromosome 21, the S100B mRNA was detected in the VZ of the mouse embryonic Cb on whole mounts and tissue sections [34].

The radial glial origin of EGFP+ S100B+ cells present in the CTZ in increasing number from E14 to E17.5 is also consistent with several reports showing that in addition to being essential for PC exit from the VZ between E13.5 and E17.5 [36], the primary radial glia is linked to gliogenesis from E14 to P7 [27]. According to ^3^H thymidine and BrdU birth dating studies, PCs are produced between E11 and E13 [37], and expression of calbindin-1 is first detectable at around E14 in post mitotic PCs that have migrated a short distance away from the VZ [26].

Here we show that EGFP+ cells that detach from the VZ and migrate into the CTZ beginning on E14.5, are not post-mitotic Purkinje cells. While migrating towards the surface most EGFP+ cells lose their connection to the VZ but not their connection to the pial membrane, a feature of prospective Bergmann glial cells. Finally, we show that near the onset of gliogenesis (E16.5), Sox9 expression is maintained in some radial glia-derived EGFP+ cells. This is in line with a previous report showing that most, if not all glial precursors in the Cb take the form of radial glial cells [27] and Bergmann glial cells maintain Sox9 expression from the early migrating progenitor stage through adulthood [29].

Several molecules known to be expressed in Bergmann glial cells of the adult mouse Cb start being expressed in the embryonic radial glia, at around E14. They include the intermediate filament protein vimentin [4], the lipid binding protein BLBP [38], the enzyme Glutamine synthetase [39], the extra cellular matrix molecule with neuron-glia cell adhesion activity Tenascin-C [40], the glutamate transporter Glast [41], the enzyme 3PGDH involved in L-serine biosynthesis [42], the Punc neural cell-adhesion molecule [43], and the transcription factor Sox9 [29]. We now suggest that S100B be added to the list.

### The midbrain-hindbrain junction: a privileged site of S100B expression before cerebellar midline fusion

The strongly S100B+ median stretch of ventral neuroepithelium we observed at the midbrain-hindbrain junction (Fig 3E) is reminiscent of a previously reported vimentin+ radial glial structure linking the IC to the Cb in E20 rat embryos [44]. In the mouse, this isthmic territory is thought to provide a cellular substratum and the signals essential for fusion of the cerebellar plates along the dorsal midline [44]. In addition, this territory is subsequently relinquished to the velum medullaris, a neuron-free sheet of cells that is very short before birth but which develops during the following days and links the anterior pole of the vermis to the inferior colliculus.

### S100B: a rhombic lip marker of primary granule precursor genesis

The RL is a specialized germinative epithelium that arises as a result of the ongoing interaction between the neural tube and the non-neural roof plate ectoderm of V4 (reviewed in [1]). Its main derivatives include primary precursors of glutamate releasing excitatory granule neurons, by far the most numerous neurons in the CNS, which relay afferent, excitatory information from mossy fibers to Purkinje neurons. In addition, the RL produces precursors of DCN neurons, the main output centers of the Cb [9], and cortical UBCs, which amplify inputs from vestibular ganglia and nuclei [45].

In the present study, we provide evidence for transcription of the *S100B *gene being specifically activated in the RL during the primary phase (E13–E17) of GP genesis. First, we have shown that expression of the *S100B*-EGFP transgene is initiated in Pax6+ precursors present in the RL at E12.5, which is after the bulk of DCN neuron precursors have left the RL [9] and reached the NTZ [8]. The non-DCN nature of the RL Pax6+ precursors is substantiated by the existence of a clear boundary, at E13.5, between the EGFP-tagged RL and the EGFP-negative NTZ (Fig 5D). Such a discontinuity is not expected to occur when using a *LacZ *reporter gene driven by the *Math1 *locus [8].

Second, we think we can exclude UBC precursors as possible candidates for *S100B*-EGFP expression on the basis of their mode of migration that is different from the subpial mode of Pax6+ EGFP+ precursor migration within the EGL. Both populations of GPs and UBC precursors are known to share expression of the RL markers Pax6 and Math1. However, unlike UBC precursors which stream from the RL and posterior EGL into the developing white matter, and are therefore excluded from the anterior EGL [7], EGFP+ Pax6+ precursors invested the entire EGL with their leading process contacting the pial surface (Fig. 5A,C), a signature of migrating GPs [44,46]. Hence, we believe that transient expression of the S100B gene is a specific marker of the primary phase of GP genesis in the embryonic RL.

Finally, although we could not detect the S100B protein in the mouse RL, evidence exists for the presence of the S100B protein in the hamster EGL [32], and S100B mRNA in the murine cerebellar VZ including the RL [12,34]. Therefore, one interpretation for our results is that both the endogenous *S100B *gene and the transgene are expressed in the RL but the S100B protein cannot be detected because its cytoplasmic level is below detection limit, or because it is released in the intercellular space. The latter cannot be expected from the reporter EGFP protein since it is not a fusion of S100B and EGFP [11].

### How is the S100B gene activated during cerebellar morphogenesis?

Before cerebellar midline fusion, the medial-most portion of the Cb neuroepithelium and abutting inferior tectal neuroepithelium, both co expressing EGFP and S100B at the highest level (Fig. 2), are also known territories of high En-1 expression [44]. This designates En-1 as a potential inducer of S100B gene expression in the Cb. Beginning on E8.5 in the mouse, the Fgf-8 molecule produced in the isthmic orgnizer [47] induces expression of a series of genes including Wnt-1 and En-1 [48]. Wnt-1 maintains En-1 expression, which in turn, positively regulates Fgf-8, resulting in amplification of the Fgf-8 signal necessary for proper Cb specification and development [49,50].

Interestingly, the earliest site of strong S100Bexpression in S100B-EGFP mice is the notochord beginning on E10.5 (Hachem and Legraverend, unpublished), and inhibition of En-1 expression by antisense targeting of early somite mouse embryos resulted in the loss of S100B expression in the notochord region subjacent to affected neural tube segments [51]. Therefore, S100B may be a downstream component of the Wnt/En-1 regulatory cascade involved in the specification of both the notochord and Cb.

### Which function(s) for S100B during cerebellar morphogenesis?

In the mouse embryonic Cb, radial glial cells and immature astrocytes express the intermediate filament vimentin [52], a protein with which S100B was shown to interact *in vitro *[53]. S100B also binds to and activates in a Ca2+-dependent manner NDR-1 and NDR-2, two nuclear proteins that belong to an evolutionary conserved subfamily of serine/threonine protein kinases involved in the regulation of cell morphology [20]. These types of interaction are consistent with S100B participating *in vivo *in the establishment and maintenance of a radial glial phenotype in the medial portion of the Cb where S100B is intracytoplasmic.

S100B may also be released from cerebellar rhombic lip EGFP+ Pax6+ precursors and promote the extension and branching of their neurites as illustrated in Fig. 6C. In 1985, Kligman and Marshak identified the extra cellular disulfide form of S100B as the molecule responsible for neurite extension of cultured chicken embryonic cortical neurons and named it "Neurite Extension Factor" [54]. This NEF effect of S100B was later demonstrated on various types of neurons including cortical neurons [55], serotonergic mesencephalic neurons [56], dorsal root ganglion neurons [57] and spinal cord neurons [58], but the mechanism responsible for the NEF effect is unknown.

Another possible role exerted by the S100B protein released in the extracellular space is that of a trophic factor. Added *in ovo *at physiological concentrations, S100B was indeed capable of preventing the naturally occurring death of chicken motor neurons [59]. The neurotrophic activity of S100B is thought to involve activation of NF-kappaB [60], binding to the receptor for advanced glycation end products (RAGE) and increased expression of the anti-apoptotic protein Bcl-2 [21].

## Methods

### Animals

Transgenic *S100B*-EGFP mice [11], and S100B knockout mice [15] were housed under standard laboratory conditions in a 12-h light/dark cycle with access to food and water *ad libitum*. Experiments were performed according to the principles of laboratory animal care, following the guidelines approved by INSERM. Adult mice were allowed to mate overnight, and females were inspected for the presence of vaginal plugs the next morning (E0.5). Pregnant females were anesthetized with sodium pentobarbital and perfused through the ascending aorta with phosphate-buffered saline (PBS, pH 7.4) followed by 300 ml of fixative composed of 4% Para formaldehyde in 0.1 M phosphate buffer, pH 7.4. Embryos were quickly removed from the uteruses, anesthetized by hypothermia, and sacrificed by decapitation.

### Immunohistochemistry

Embryos were cryoprotected by immersion in 30% sucrose in PBS for 12–24 h at 4°C, embedded in OCT compound (Tissue-Tek, Washington, DC) and frozen at -30°C. Tissue sections (15 μM) were obtained in a JUNG CM 300 cryostat (Leica), mounted onto poly-lysine-coated slides, and stored at -20°C. After several rinses in PBS, frozen sections were incubated in PBS containing 0.1% Triton X-100 and 10% goat serum for 1 h at room temperature (RT) and then incubated for 24 h at 4°C with primary antibodies followed by incubation with secondary antibodies for 1 h at RT. Primary antibodies were diluted in PBS containing 0.1% Triton X-100 and secondary antibodies were diluted in PBS containing 0.05% bovine serum albumin (BSA). After three rinses in PBS, sections were mounted in Mowiol (Calbiochem, La Jolla, CA) containing 2.5% 1,4-diazabicyclo- (2.2.2) octane (DABCO). The specificity of S100B immunolabeling was confirmed on sections of S100B null embryos (data not shown).

### Primary and secondary antibodies

The following antibodies were used: rabbit polyclonal antibodies specific for S100B (1:1,000; Dako, Glostrup, Denmark, or Carpinteria, CA); Olig2 (1:6,000; obtained from Dr. Takebayashi, National Institute for Physiological Sciences, Okazaki, Japan); Pax6 (1:500; Berkeley Antibody Company, Richmond CA); Sox9 (1:500; Santa Cruz Biotechnology, Santa Cruz, CA); calbindin-1 (1:1000; Swant, Bellinzona, Switzerland). We used secondary donkey anti-rabbit IgG Cy3-F (ab0) 2 conjugates (1:1,000; Jackson ImmunoResearch, West Grove, PA). The specificity of S100B immunolabeling was confirmed on brain sections of S100B knockout embryos.

### Confocal microscopy and illustrations

A Bio-Rad 1024 confocal laser scanning microscopic (CLSM) system equipped with an argon/krypton mixed gas laser was used for the analysis of adult and embryonic S100B-EGFP mice. Non-stacked CLSM images were obtained, representing optical sections with a depth of field of 0.5–3 μM. Two laser lines emitting at 488 nm and 568 nm were used for exciting EGFP and CY3-conjugated secondary antibodies, respectively. Four inputs were averaged to reduce the background noise, and green and red images were collected sequentially. Data acquisition and processing were controlled by the Laser sharp 1024 software and processing system. Unless otherwise stated, we marked EGFP+ Pax6- cells with asterisks and EGFP+ Pax6+ double positives with colored dots (green over red pax6+ nuclei, red over green EGFP+ cells). Pax6+ EGFP- cells were left unmarked. A cell was considered EGFP+ when both nucleus and cytoplasm were stained, or if a rim of green cytoplasm could be clearly assigned to a given nucleus. As a result, the number of double positives was most probably under evaluated. The pial and ventricular surfaces were outlined with white dashed lines; the limit between neuroepithelium and choroid plexus epithelium was marked with a blue dashed line; the choroid plexus epithelium of the fourth ventricle was outlined with a red dashed line.

## Abbreviations

**V **trigeminal ganglion

**VII **facial ganglion

**ant **anterior

**aq **aqueduct

**BLBP **brain lipid binding protein

**cap **blood capillary

**Cb **cerebellum

**CNS **central nervous system

**CP **chroroid plexus of the fourth ventricle

**Cpe **choroid plexus epithelium

**CTZ **cortical transitory zone

**DCN **deep cerebellar nuclei

**di **diencephalon

**dors **dorsal

**EGFP **enhanced green fluorescent protein

**egl **external granule layer

**En-1 **engrailed 1

**Fgf-8 **fibroblast growth factor 8

**Gap43 **growth-associated protein 43

**GFAP **glial fibrillary acidic protein

**GP **cerebellar granule neuron precursor

**IC **inferior colliculus

**lat **lateral

**med **medial

**mes **mesencephalon

**NDR **nuclear (Saccharomyces cerevisiae) Dbf2-related kinase

**NTZ **nuclear transitory zone

**PC **Purkinje cells

**Post **posterior

**RL **cerebellar rhombic lip

**scc **semi-circular canal of otic vesicle

**tel **telencephalon

**UBC **unipolar brush cells

**V4 **fourth ventricle

**VZ **ventricular zone

**Wnt-1 **wingless

## Authors' contributions

SH carried out the immunoassays and confocal microscopy. A-SL carried out the Pax6 immunoassay. J-PH participated in the design of the study. CL conceived of the study, designed and coordinated it and drafted the manuscript. All authors read and approved the final manuscript.

## References

[B1] Joyner AL, Zervas M (2006). Genetic inducible fate mapping in mouse: Establishing genetic lineages and defining genetic neuroanatomy in the nervous system. Dev Dyn.

[B2] Altman J, Bayer SA (1985). Embryonic development of the rat cerebellum. III. Regional differences in the time of origin, migration, and settling of Purkinje cells. J Comp Neurol.

[B3] Ben-Arie N, Bellen HJ, Armstrong DL, McCall AE, Gordadze PR, Guo Q, Matzuk MM, Zoghbi HY (1997). Math1 is essential for genesis of cerebellar granule neurons. Nature.

[B4] Kamei Y, Inagaki N, Nishizawa M, Tsutsumi O, Taketani Y, Inagaki M (1998). Visualization of mitotic radial glial lineage cells in the developing rat brain by Cdc2 kinase-phosphorylated vimentin. Glia.

[B5] Yuasa S (1996). Bergmann glial development in the mouse cerebellum as revealed by tenascin expression. Anat Embryol (Berl).

[B6] Machold R, Fishell G (2005). Math1 is expressed in temporally discrete pools of cerebellar rhombic-lip neural progenitors. Neuron.

[B7] Englund C, Kowalczyk T, Daza RA, Dagan A, Lau C, Rose MF, Hevner RF (2006). Unipolar brush cells of the cerebellum are produced in the rhombic lip and migrate through developing white matter. J Neurosci.

[B8] Wang VY, Rose MF, Zoghbi HY (2005). Math1 expression redefines the rhombic lip derivatives and reveals novel lineages within the brainstem and cerebellum. Neuron.

[B9] Fink AJ, Englund C, Daza RA, Pham D, Lau C, Nivison M, Kowalczyk T, Hevner RF (2006). Development of the deep cerebellar nuclei: transcription factors and cell migration from the rhombic lip. J Neurosci.

[B10] Engelkamp D, Rashbass P, Seawright A, van Heyningen V (1999). Role of Pax6 in development of the cerebellar system. Development.

[B11] Vives V, Alonso G, Solal AC, Joubert D, Legraverend C (2003). Visualization of S100B-positive neurons and glia in the central nervous system of EGFP transgenic mice. J Comp Neurol.

[B12] Landry CF, Ivy GO, Dunn RJ, Marks A, Brown IR (1989). Expression of the gene encoding the beta-subunit of S-100 protein in the developing rat brain analyzed by in situ hybridization. Brain Res Mol Brain Res.

[B13] Zimmer DB, Chaplin J, Baldwin A, Rast M (2005). S100-mediated signal transduction in the nervous system and neurological diseases. Cell Mol Biol (Noisy-le-grand).

[B14] Jiang H, Shah S, Hilt DC (1993). Organization, sequence, and expression of the murine S100 beta gene. Transcriptional regulation by cell type-specific cis-acting regulatory elements. J Biol Chem.

[B15] Xiong Z, O'Hanlon D, Becker LE, Roder J, MacDonald JF, Marks A (2000). Enhanced Calcium Transients in Glial Cells in Neonatal Cerebellar Cultures Derived from S100B Null Mice. Exp Cell Res.

[B16] Nishiyama H, Takemura M, Takeda T, Itohara S (2002). Normal development of serotonergic neurons in mice lacking S100B. Neurosci Lett.

[B17] Ralay Ranaivo H, Craft JM, Hu W, Guo L, Wing LK, Van Eldik LJ, Watterson DM (2006). Glia as a therapeutic target: selective suppression of human amyloid-beta-induced upregulation of brain proinflammatory cytokine production attenuates neurodegeneration. J Neurosci.

[B18] Ivanenkov VV, Jamieson GA, Gruenstein E, Dimlich RV (1995). Characterization of S-100b binding epitopes. Identification of a novel target, the actin capping protein, CapZ. J Biol Chem.

[B19] Wilder PT, Lin J, Bair CL, Charpentier TH, Yang D, Liriano M, Varney KM, Lee A, Oppenheim AB, Adhya S, Carrier F, Weber DJ (2006). Recognition of the tumor suppressor protein p53 and other protein targets by the calcium-binding protein S100B. Biochim Biophys Acta.

[B20] Stegert MR, Tamaskovic R, Bichsel SJ, Hergovich A, Hemmings BA (2004). Regulation of NDR2 protein kinase by multi-site phosphorylation and the S100B calcium-binding protein. J Biol Chem.

[B21] Huttunen HJ, Kuja-Panula J, Sorci G, Agneletti AL, Donato R, Rauvala H (2000). Coregulation of neurite outgrowth and cell survival by amphoterin and S100 proteins through receptor for advanced glycation end products (RAGE) activation. J Biol Chem.

[B22] Schafer BW, Heizmann CW (1996). The S100 family of EF-hand calcium-binding proteins: functions and pathology. Trends Biochem Sci.

[B23] Donato R (2001). S100: a multigenic family of calcium-modulated proteins of the EF-hand type with intracellular and extracellular functional roles. Int J Biochem Cell Biol.

[B24] Arcuri C, Bianchi R, Brozzi F, Donato R (2005). S100B increases proliferation in PC12 neuronal cells and reduces their responsiveness to nerve growth factor via Akt activation. J Biol Chem.

[B25] Frizzo JK, Tramontina AC, Tramontina F, Gottfried C, Leal RB, Donato R, Goncalves CA (2004). Involvement of the S100B in cAMP-induced cytoskeleton remodeling in astrocytes: a study using TRTK-12 in digitonin-permeabilized cells. Cell Mol Neurobiol.

[B26] Yuasa S, Kawamura K, Ono K, Yamakuni T, Takahashi Y (1991). Development and migration of Purkinje cells in the mouse cerebellar primordium. Anat Embryol (Berl).

[B27] Yamada K, Watanabe M (2002). Cytodifferentiation of Bergmann glia and its relationship with Purkinje cells. Anat Sci Int.

[B28] Hartfuss E, Galli R, Heins N, Gotz M (2001). Characterization of CNS precursor subtypes and radial glia. Dev Biol.

[B29] Pompolo S, Harley VR (2001). Localisation of the SRY-related HMG box protein, SOX9, in rodent brain. Brain Res.

[B30] Hashimoto M, Mikoshiba K (2003). Mediolateral compartmentalization of the cerebellum is determined on the "birth date" of Purkinje cells. J Neurosci.

[B31] Yamada K, Fukaya M, Shibata T, Kurihara H, Tanaka K, Inoue Y, Watanabe M (2000). Dynamic transformation of Bergmann glial fibers proceeds in correlation with dendritic outgrowth and synapse formation of cerebellar Purkinje cells. J Comp Neurol.

[B32] Sievers J, Pehlemann FW, Gude S, Hartmann D, Berry M (1994). The development of the radial glial scaffold of the cerebellar cortex from GFAP-positive cells in the external granular layer. J Neurocytol.

[B33] Takebayashi H, Ohtsuki T, Uchida T, Kawamoto S, Okubo K, Ikenaka K, Takeichi M, Chisaka O, Nabeshima Y (2002). Non-overlapping expression of Olig3 and Olig2 in the embryonic neural tube. Mech Dev.

[B34] Reymond A, Marigo V, Yaylaoglu MB, Leoni A, Ucla C, Scamuffa N, Caccioppoli C, Dermitzakis ET, Lyle R, Banfi S, Eichele G, Antonarakis SE, Ballabio A (2002). Human chromosome 21 gene expression atlas in the mouse. Nature.

[B35] Human chromosome 21 genes and murine orthologues.. http://www.tigem.it/ch21exp/body/bodyS100B.html.

[B36] Yuasa S, Kitoh J, Oda S, Kawamura K (1993). Obstructed migration of Purkinje cells in the developing cerebellum of the reeler mutant mouse. Anat Embryol (Berl).

[B37] Miale IL, Sidman RL (1961). An autoradiographic analysis of histogenesis in the mouse cerebellum. Exp Neurol.

[B38] Feng L, Hatten ME, Heintz N (1994). Brain lipid-binding protein (BLBP): a novel signaling system in the developing mammalian CNS. Neuron.

[B39] Reichenbach A, Siegel A, Rickmann M, Wolff JR, Noone D, Robinson SR (1995). Distribution of Bergmann glial somata and processes: implications for function. J Hirnforsch.

[B40] Yuasa S, Kawamura K, Kuwano R, Ono K (1996). Neuron-glia interrelations during migration of Purkinje cells in the mouse embryonic cerebellum. Int J Dev Neurosci.

[B41] Watase K, Hashimoto K, Kano M, Yamada K, Watanabe M, Inoue Y, Okuyama S, Sakagawa T, Ogawa S, Kawashima N, Hori S, Takimoto M, Wada K, Tanaka K (1998). Motor discoordination and increased susceptibility to cerebellar injury in GLAST mutant mice. Eur J Neurosci.

[B42] Yamasaki M, Yamada K, Furuya S, Mitoma J, Hirabayashi Y, Watanabe M (2001). 3-Phosphoglycerate dehydrogenase, a key enzyme for l-serine biosynthesis, is preferentially expressed in the radial glia/astrocyte lineage and olfactory ensheathing glia in the mouse brain. J Neurosci.

[B43] Yang W, Li C, Mansour SL (2001). Impaired motor coordination in mice that lack punc. Mol Cell Biol.

[B44] Louvi A, Alexandre P, Metin C, Wurst W, Wassef M (2003). The isthmic neuroepithelium is essential for cerebellar midline fusion. Development.

[B45] Nunzi MG, Birnstiel S, Bhattacharyya BJ, Slater NT, Mugnaini E (2001). Unipolar brush cells form a glutamatergic projection system within the mouse cerebellar cortex. J Comp Neurol.

[B46] Jensen P, Smeyne R, Goldowitz D (2004). Analysis of cerebellar development in math1 null embryos and chimeras. J Neurosci.

[B47] Chi CL, Martinez S, Wurst W, Martin GR (2003). The isthmic organizer signal FGF8 is required for cell survival in the prospective midbrain and cerebellum. Development.

[B48] Joyner AL, Liu A, Millet S (2000). Otx2, Gbx2 and Fgf8 interact to position and maintain a mid-hindbrain organizer. Curr Opin Cell Biol.

[B49] Hidalgo-Sanchez M, Millet S, Simeone A, Alvarado-Mallart RM (1999). Comparative analysis of Otx2, Gbx2, Pax2, Fgf8 and Wnt1 gene expressions during the formation of the chick midbrain/hindbrain domain. Mech Dev.

[B50] Hidalgo-Sanchez M, Simeone A, Alvarado-Mallart RM (1999). Fgf8 and Gbx2 induction concomitant with Otx2 repression is correlated with midbrain-hindbrain fate of caudal prosencephalon. Development.

[B51] Augustine KA, Liu ET, Sadler TW (1995). Antisense inhibition of engrailed genes in mouse embryos reveals roles for these genes in craniofacial and neural tube development. Teratology.

[B52] Bovolenta P, Liem RK, Mason CA (1984). Development of cerebellar astroglia: transitions in form and cytoskeletal content. Dev Biol.

[B53] Sorci G, Agneletti AL, Bianchi R, Donato R (1998). Association of S100B with intermediate filaments and microtubules in glial cells. Biochim Biophys Acta.

[B54] Kligman D, Marshak DR (1985). Purification and characterization of a neurite extension factor from bovine brain. Proc Natl Acad Sci U S A.

[B55] Winningham-Major F, Staecker JL, Barger SW, Coats S, Van Eldik LJ (1989). Neurite extension and neuronal survival activities of recombinant S100 beta proteins that differ in the content and position of cysteine residues. J Cell Biol.

[B56] Azmitia EC, Dolan K, Whitaker-Azmitia PM (1990). S-100B but not NGF, EGF, insulin or calmodulin is a CNS serotonergic growth factor. Brain Res.

[B57] Van Eldik LJ, Christie-Pope B, Bolin LM, Shooter EM, Whetsell WO (1991). Neurotrophic activity of S-100 beta in cultures of dorsal root ganglia from embryonic chick and fetal rat. Brain Res.

[B58] Nishi M, Kawata M, Azmitia EC (1997). S100beta promotes the extension of microtubule associated protein2 (MAP2)-immunoreactive neurites retracted after colchicine treatment in rat spinal cord culture. Neurosci Lett.

[B59] Bhattacharyya A, Oppenheim RW, Prevette D, Moore BW, Brackenbury R, Ratner N (1992). S100 is present in developing chicken neurons and Schwann cells and promotes motor neuron survival in vivo. J Neurobiol.

[B60] Alexanian AR, Bamburg JR (1999). Neuronal survival activity of s100betabeta is enhanced by calcineurin inhibitors and requires activation of NF-kappaB. Faseb J.

